# A Dictionary of Terms Used in Medical and the Collateral Sciences

**Published:** 1856-01

**Authors:** 


*A Dictionary of Terms used in Medical and the Collateral Sciences:* by Richard D. Hoblyn, A. M., Oxn. A new American, from the last London, edition—revised, with numerous additions, by Isaac Hays, M. D., Editor of the American Journal of the Medical Sciences. Philadelphia: Blanchard & Lea, 1855.

This is a convenient little work for reference, and will, we have no doubt, continue to be a favorite with both the student and physician. Dr. Hays has adapted it to the present condition of Medical Science, by the incorporation of all those terms which have recently been brought into use.

For sale by Keen & Lee. J.

